# Addressing inequities in the otolaryngology academic publishing: A call to action

**DOI:** 10.4102/jcmsa.v2i1.64

**Published:** 2024-06-11

**Authors:** Sarah Nuss, Rolvix H. Patterson, Mary J. Xu, Amina Seguya, Valerie Salano, Nader Zalaquett, Samuel Okerosi, Bethany Hedt-Gauthier, Johannes Fagan

**Affiliations:** 1 Global OHNS Initiative, Durham, United States of America; 2 Warren Alpert Medical School, Brown University, Providence, United States of America; 3 Department of Head and Neck Surgery and Communication Sciences, Duke University School of Medicine, Durham, United States of America; 4 Department of Otolaryngology, Head and Neck Surgery, University of California, San Francisco, United States of America; 5 Department of Otolaryngology, Head and Neck Surgery, Mulago National Referral Hospital, Kampala, Uganda; 6 Department of Otorhinolaryngology, Nyahururu County Hospital, Nyahururu, Kenya; 7 American University of Beirut, Beirut, Lebanon; 8 Department of Otolaryngology, Machakos Level Five Hospital, Machakos, Kenya; 9 Department of Global Health and Social Medicine, Harvard Medical School, Boston, United States of America; 10 Division of Otolaryngology, University of Cape Town, Cape Town, South Africa

**Keywords:** global surgery, global otolaryngology, research equity, article processing charges, open access

## Abstract

Article processing charges (APCs) for open access (OA) journals perpetuate inequities in scientific knowledge. High APCs systematically restrict low- and middle-income country (LMIC) researchers from contributing to research knowledge, preventing the dissemination of high-value, high-quality, and sustainable LMIC-driven solutions. Otolaryngology journals are no exception. The authors propose solutions to rectify the inequities in academic publishing because of APCs, including innovative solutions adopted by several major journals. Addressing these inequities requires medical society and journal editorial board leadership to ensure equitable APC policies.

## Background

Collaboration between high-income countries (HICs) and low- and middle-income countries (LMICs) is crucial for advancements in global otolaryngology-head and neck surgery (OHNS). However, in these collaborations, benefits such as research recognition and career advancement are often skewed in favour of HIC researchers. While the problem of inequity in global health research collaborations is multifaceted, a key barrier to research dissemination for LMIC authors is the article processing charges (APC) model for open access (OA) journals.

Open access publishing makes research available without subscription or paywall barriers. While OA improves access to research, components of this system continue to perpetuate inequity. Open access gained popularity in the early 2000s when the National Institutes for Health (NIH) mandated that NIH-supported research must be freely available within 1 year of publication.^1^ This requirement prompted a shift in the financing model for scientific journals. Rather than relying on income from user subscriptions, many journals implemented the APC model, which relies on individual authors, institutions, or consortia to cover publication overhead and operational costs.

This model imposes challenges for researchers who lack grant funding or institutional support. These researchers must decide whether it is worthwhile, or even feasible, to pay for the APC to make their research findings freely available. Authors may choose to pay APCs using their personal finances or funding that would otherwise go towards additional research. Given the high variability and lack of cost transparency in APCs, it is often unclear what portion of these fees represents publication costs versus profit.^2,3^ While initiatives such as the Health Inter Network Access to Research Initiative (HINARI) of the World Health Organization (WHO) were founded to enable LMICs’ access to medical literature at reduced subscription costs, these initiatives target the pay-to-view model, and fail to address the recent shift to the pay-to-publish model.

Article processing charges can create barriers for LMIC researchers who aim to contribute to scientific knowledge. While many journals offer LMIC waivers, they are not always effective in increasing publishing access for LMIC authors.^4^ Waivers are typically applied only on request, and a study by Burchardt et al.^5^ found that only four (15%) of the world’s largest OA publishers offered automatic waivers.^5,6^ There is increasing evidence to suggest that LMIC researchers who are not able to pay APCs are not utilising these waivers.^4^ While some OA otolaryngology journals offer automatic waivers to LMIC authors, there are nuances to waiver lists. These waivers often exclude researchers from middle-income countries and authors who are part of HIC/LMIC collaborations. For example, journal waivers may require that either all authors or > 50% of authors are from an LMIC. Furthermore, journals with the highest APCs, which are often North American journals with higher impact factors, are less likely to offer waivers to LMIC authors.^7^

The OA model is common in otolaryngology journals, and a recent review found that most authors represented in OA otolaryngology journals are based in HICs.^8^ Only 33% of OA otolaryngology journals offer APC subsidies for LMIC authors.^2^ Article processing charges fluctuate significantly across the five highest impact factor otolaryngology journals, ranging from $1430.00 to $5000.00. Among OA otolaryngology journals, 77% require APCs, with an average cost of $2452.00.^2^ These fees are high relative to income in many regions of the world. For example, the annual Gross National Income (GNI) per capita is $3930.00 Purchasing Power Parity in sub-Saharan Africa.^9^ A recent study underscored the challenge, finding that 87% of LMIC authors earned less than $1500.00 per month and 52% of trainees did not receive any salary.^10^

Despite the financial challenges posed by APCs, there are substantial barriers to reducing these fees. The APCs are essential for covering operating costs. They also represent a significant source of revenue for publishers, which may be an important target to prioritise in considering financing mechanisms to support LMIC authors.

## Addressing inequity in models for article processing charges

Here, we propose solutions to address the inequities in academic publishing related to APCs. Several major scientific journals outside of otolaryngology have recognised the ethical responsibility to ensure equitable APC policies by adopting innovative solutions. For example, journals may offer reduced or no-cost publishing for LMIC authors. Additionally, they may implement a graded APC requirement that accounts for country Gross Domestic Product, Human Development Index, or Institutional Direct Cost rates. Implementation of these policies may vary by journal; for example, some determine the classification based on the academic affiliation of the first or corresponding author.

Article processing charge waivers may be granted based on the availability of resources rather than country of affiliation. For example, the Colleges of Medicine of South Africa’s new multidisciplinary OA journal charges zero APCs for authors without institutional or grant support. Otolaryngology journals should strongly consider adopting this equity-centred publishing strategy. In addition, journals should consider offering automatic fee waivers for LMIC authors. Finally, in the absence of full fee waivers, journals should consider setting aside a scholarship fund to regularly sponsor APCs for LMIC-authored articles.

To finance these solutions, journal leadership and medical societies must lobby for institutional financial support or seek to partner with independent publishers. Collaborations with private sponsors such as foundations, corporations or research institutions can also be pursued to create innovative funding mechanisms. For example, establishing consortia funding to support diamond OA models, which do not burden the authors with charges, is a viable alternative framework. In addition, pooling resources to establish a fund to support LMIC authors may be an impactful approach.

Furthermore, there is a need for transparency around publishing costs. Research funding organisations are mobilising to support immediate, accessible OA publishing while ensuring cost transparency.^11^ Enhanced insight into the costs and profits associated with OA publishing can guide targeted interventions to address APC barriers for LMIC researchers.

Researchers and publishers in otolaryngology have a shared responsibility to promote equitable access to knowledge. The APCs are a priority area for intervention to promote LMIC-led research and dissemination. By reducing the burden of APCs for LMIC researchers, we can expand access to knowledge and ultimately improve healthcare outcomes around the world.

## Conclusion

The APC model poses challenges to both publishing and accessing research findings, depriving the world of valuable insights and diverse perspectives. Low- and middle-income countries have a disproportionate burden of OHNS disease, and high OA publishing fees can prevent the dissemination of LMIC-driven solutions to these disparities.^1^ Furthermore, studies have demonstrated that research conducted in LMICs often does not align with national health priorities.^1^ Without increased equity in both the production and accessibility of scientific knowledge, the most vulnerable patients will continue to suffer preventable illness and death.

## References

[1] An open access mandate for the National Institutes of Health – PMC [homepage on the Internet]. [cited 2023 Mar 1]. Available from: https://www.ncbi.nlm.nih.gov/pmc/articles/PMC3090178/.

[2] Kim EK, Shrime MG. Cost of open access publishing in otolaryngology-head and neck surgery. World J Otorhinolaryngol – Head Neck Surg. 2022;9(4):78. 10.1002/wjo2.78 PMC10696265 38059140

[3] Budzinski O, Grebel T, Wolling J, Zhang X. Drivers of article processing charges in open access. Scientometrics. 2020;124(3):2185–2206. 10.1007/s11192-020-03578-3

[4] Smith E, Haustein S, Mongeon P, Shu F, Ridde V, Larivière V. Knowledge sharing in global health research – The impact, uptake and cost of open access to scholarly literature. Health Res Policy Syst. 2017;15(1):73. 10.1186/s12961-017-0235-3 28851401 PMC5576373

[5] Burchardt J. Researchers outside APC-financed open access: Implications for scholars without a paying institution. SAGE Open. 2014;4(4):2158244014551714. 10.1177/2158244014551714

[6] Klebel T, Ross-Hellauer T. The APC-barrier and its effect on stratification in open access publishing. Quant Sci Stud. 2023;4(1):22–43. 10.1162/qss_a_00245

[7] Gardner UG, Thompson PS, Burton J, Stewart C, Fuller CD, Rooney MK, et al. Article Processing Charge Waiver Policies as a Barrier to Oncology Scholarship in Low- and Lower-Middle-Income Countries. JCO Glob Oncol. 2021 Sep 23;7:GO.21.00143. 34554813 10.1200/GO.21.00143 PMC8478385

[8] Jashek-Ahmed F, Daudu D, Heer B, et al. Female and low- and middle-income authorship trends in high-impact ENT journals (2011–2020). Laryngoscope Investig Otolaryngol. 2021;7:1413–1417. 10.1002/lio2.1044 PMC10116964 37090877

[9] The World Bank. GNI per capita, PPP (current international $) – Sub-Saharan Africa [homepage on the Internet]. [cited 2023 Mar 1]. Available from: https://data.worldbank.org/indicator/NY.GNP.PCAP.PP.CD?end=2021&locations=ZG&start=2021.

[10] Seguya A, Salano V, Okerosi S, et al. Are open access article processing charges affordable for otolaryngologists in low-income and middle-income countries?. Curr Opin Otolaryngol Head Neck Surg. 2023;31(3):202–207. 10.1097/moo.0000000000000892 37144583

[11] Plan S Principles | Plan S [homepage on the Internet]. [cited 2023 Jun 29]. Available from: https://www.coalition-s.org/plan_s_principles/.

